# Informational masking modulates semantic processing during recognition of syntactically complex speech: evidence from pupillometry

**DOI:** 10.3389/fpsyg.2026.1926700

**Published:** 2026-09-18

**Authors:** Jing Shen, Gayle DeDe

**Affiliations:** Department of Communication Sciences and Disorders, Temple University, Philadelphia, PA, United States

**Keywords:** informational masking, listening effort, pupillometry, semantic processing, sentence processing, speech perception

## Abstract

**Introduction:**

Speech perception in adverse listening environments depends not only on the quality of the acoustic signal but also on listeners’ ability to recruit higher-level linguistic information. Although semantic constraints are known to influence recognition of syntactically complex speech, it remains unclear how informational masking, in which competing speech interferes with target-speech processing beyond peripheral acoustic overlap, influences the use of these cues during spoken sentence processing. The present study examined the effects of animacy-based semantic constraints on recognition of subject- and object-relative sentences presented in either an original two-talker speech masker or a time-reversed version of the same masker.

**Methods:**

Thirty-seven young adults with normal hearing completed a sentence repetition task while pupil size was recorded continuously. Speech recognition was analyzed using generalized linear mixed-effects models, and pupil responses were analyzed using generalized additive mixed models (GAMMs).

**Results:**

Consistent with previous findings, semantic effects were substantially greater for syntactically complex object-relative sentences than for subject-relative sentences. Masker type did not produce a significant overall effect on recognition accuracy, nor did it significantly modify the behavioral semantic effect. In the pupil data, however, semantic condition interacted significantly with masker type: the magnitude and direction of the animacy effect differed between the original- and reversed-speech masker conditions, whereas the corresponding time-varying interaction did not reach significance.

**Discussion:**

These findings suggest that informational masking alters how semantic information contributes to processing demands during sentence recognition rather than simply increasing overall listening effort. More broadly, the results demonstrate the value of combining behavioral and pupillometric measures to examine how perceptual and linguistic demands jointly shape spoken language processing.

## Introduction

Successful speech perception in everyday environments requires listeners to integrate incoming acoustic information with linguistic knowledge. Although traditional speech-perception research has focused heavily on recognition of phonemes and words, more recent evidence emphasizes that spoken language understanding depends on interactions among perceptual, cognitive, and linguistic processes operating across multiple levels of representation (Rönnberg et al., 2013; Wingfield et al., 2015; Pichora-Fuller et al., 2016). It is well established that, when the speech signal is degraded by background noise or competing speech, listeners increasingly rely on higher-level linguistic information to support recognition and comprehension. In natural communication, visual speech cues, including facial and articulatory movements, can provide an additional source of information for listeners with normal hearing as well as those with hearing impairment when auditory input is degraded (Chandrasekaran et al., 2009; Moradi et al., 2017). At the same time, adverse listening conditions can increase perceptual demands and reduce the cognitive resources available for subsequent speech processing.

### Linguistic cues in speech perception under adverse conditions

A growing body of research demonstrates that linguistic information influences speech perception under adverse listening conditions. Speech perception in adverse listening environments depends not only on the acoustic signal but also on listeners’ ability to use higher-level linguistic information. Lexical, semantic, syntactic, and prosodic cues can facilitate word recognition and sentence understanding, particularly when acoustic information is degraded or distorted by background noise (Hoen et al., 2007; Golestani et al., 2009; Wendt et al., 2016; Jesse and Helfer, 2019; Fitzgerald et al., 2024; Shen and DeDe, 2025). During online sentence processing, listeners continuously integrate these sources of information to generate predictions about upcoming speech and to resolve linguistic ambiguity as the speech signal unfolds (Weber et al., 2006; DeDe, 2010). When listening conditions are challenging, reliance on such linguistic cues may increase, particularly for sentences with greater syntactic complexity (Van Os et al., 2022; Hsin et al., 2023; Shen and DeDe, 2025). These effects are consistent with interactive accounts of speech perception, in which higher-level linguistic knowledge contributes to efficient interpretation of degraded speech signals.

The interaction between linguistic processing and adverse listening conditions is particularly important in environments containing competing speech, which leads to informational masking. This masking condition is of great clinical interest as it represents many real-life communication challenges with speech maskers. Energetic masking occurs when acoustic overlap between the target and masker reduces the audibility of the target signal at the peripheral auditory level. Informational masking, in contrast, refers to additional interference that arises when competing sounds—particularly intelligible speech—engage perceptual or cognitive processes beyond this peripheral overlap (Freyman et al., 2004; Kidd et al., 2008; Kidd and Colburn, 2017). Previous studies have shown that informational masking can alter listeners’ ability to use linguistic cues during speech perception and increase the cognitive resources required for successful comprehension (Mattys et al., 2012; Pichora-Fuller et al., 2016). Together, these findings suggest that understanding speech in complex environments depends not only on the quality of the acoustic signal but also on how listeners use higher-level linguistic information to compensate for perceptual uncertainty and cognitive interference.

Syntactically complex speech provides a particularly useful framework for examining interactions between perceptual and linguistic processing during speech perception. Unlike isolated words or simple sentences, complex sentence structures require listeners to maintain and integrate information across multiple words while continuously assigning grammatical and thematic relationships among sentence constituents. Successful recognition of syntactically complex speech therefore depends not only on accurate perception of the acoustic signal but also on the efficient use of higher-level linguistic processes, including syntactic parsing, semantic integration, and working-memory resources (Just and Carpenter, 1992; Gibson, 1998; Wingfield et al., 2006). Consequently, perceptual degradation may have effects that extend beyond word recognition and influence the ability to establish accurate, meaningful sentence interpretations.

The interaction between syntactic complexity and adverse listening conditions has received increasing attention in both psycholinguistic and hearing-science research. Previous studies have shown that complex sentence structures, such as object-relative clauses and semantically reversible sentences, are more difficult to recognize and comprehend than simpler sentence structures, particularly under challenging listening conditions (Traxler et al., 2002; Wingfield et al., 2006; Tun et al., 2010; DeDe, 2015). These findings suggest that adverse listening conditions may disproportionately affect linguistic processes that require listeners to maintain multiple interpretations, resolve ambiguities, and assign thematic roles during online sentence processing. As a result, syntactically complex speech provides an ecologically valid and theoretically informative context for examining how listeners compensate for perceptual uncertainty during spoken language processing.

### Effects of animacy in processing of syntactic complexity

Listeners can recruit multiple linguistic cues to support processing of syntactically complex speech. Previous research has demonstrated important contributions from lexical-semantic information, discourse context, and prosodic cues in guiding sentence interpretation and resolving structural ambiguity (MacDonald et al., 1994; Cutler et al., 1997; Mattys et al., 2012). A central challenge during processing of syntactically complex sentences is thematic-role assignment, or determining “who did what to whom” within a sentence. Accurate thematic-role assignment requires listeners to continuously integrate syntactic structure with semantic information as the sentence unfolds, particularly when multiple interpretations are temporarily possible. Consequently, listeners often rely on linguistic cues that can facilitate assignment of agent and patient roles during online speech processing.

In thematic-role assignment, a particularly powerful cue is animacy. Because animate entities are more likely to serve as agents of actions (“who”) and inanimate entities are more likely to serve as patients (“to whom”), listeners rapidly use animacy information to generate expectations about thematic roles during sentence comprehension. These expectations are thought to reflect probabilistic regularities acquired through language experience and world knowledge (MacDonald et al., 1994; Altmann and Kamide, 1999), whereby certain semantic relationships occur more frequently than others in everyday communication. As a result, listeners often use animacy as a semantic cue to guide thematic-role assignment and anticipate “who did what to whom”, before all linguistic information has been fully processed. Such predictive processing can facilitate speech processing when animacy cues are consistent with the intended sentence interpretation, but it can also interfere with processing when animacy cues bias listeners toward an incorrect thematic-role assignment. These expectations are especially influential in syntactically complex sentences, where successful interpretation requires integration of semantic and syntactic information. Previous psycholinguistic studies have shown that animacy influences online sentence processing, particularly for syntactically complex object-relative sentences, by facilitating thematic-role assignment during incremental comprehension (Weckerly and Kutas, 1999; Mak et al., 2002; Traxler et al., 2002).

Object-relative clauses provide a useful framework for examining how listeners integrate syntactic structure and semantic information during thematic-role assignment. Compared with subject-relative clauses, object-relative clauses place greater demands on online sentence processing because listeners must maintain and revise interpretations as the sentence unfolds. Object-relative clauses are especially sensitive to semantic constraints because animacy information can either support or conflict with the interpretation required by the sentence structure. In particular, animacy information can either support or conflict with the thematic-role assignments implied by sentence structure, making object-relative clauses especially sensitive to the influence of semantic expectations. Using eye-tracking during reading, Traxler et al. (2002) demonstrated that animacy strongly influences processing of object-relative clauses, with increased processing costs observed when animacy-based expectations conflict with the intended thematic-role assignment. These findings suggest that sentence processing relies on continuous interactions between syntactic and semantic information, rather than syntactic structure alone, as listeners incrementally construct sentence meaning.

Although these effects have been studied extensively in psycholinguistic investigations of language processing, relatively few studies have examined whether similar mechanisms influence spoken sentence recognition. Extending this work to speech perception, DeDe (2015) examined recognition of syntactically complex spoken sentences that varied in the extent to which animacy cues supported thematic-role assignment. The results demonstrated that animacy significantly influenced sentence recognition performance, particularly for object-relative sentences. Importantly, these findings suggest that semantic constraints continue to influence processing even after listeners have successfully identified the acoustic speech signal, highlighting the contribution of higher-level linguistic processes to spoken sentence recognition.

### Animacy effects in speech recognition under informational masking

Previous research has demonstrated that semantic constraints derived from animacy influence both language processing and spoken sentence recognition. Psycholinguistic studies have shown robust animacy effects during the processing of syntactically complex sentences, particularly object-relative clauses (Mak et al., 2002; Traxler et al., 2002). Similarly, DeDe (2015) demonstrated that animacy influences recognition of spoken object-relative sentences, suggesting that semantic constraints continue to shape sentence processing after acoustic information has been successfully identified. However, most psycholinguistic investigations of animacy effects have employed reading paradigms, whereas studies of spoken sentence recognition have largely examined performance in quiet listening conditions. Consequently, relatively little is known about how listeners use semantic constraints during recognition of syntactically complex speech when the speech signal is degraded by competing speech that poses informational masking. One approach to manipulating informational masking is to compare intelligible competing speech with time-reversed speech. Time reversal preserves many of the acoustic characteristics of the speech masker while substantially reducing its linguistic intelligibility, thereby reducing interference from competing linguistic information. Thus, comparing original and time-reversed speech maskers provides a means of examining how the presence of intelligible competing speech influences the use of semantic constraints during target-sentence recognition.

Existing theoretical frameworks suggest at least two competing hypotheses regarding the effects of informational masking on the use of semantic constraints. One possibility, consistent with resource-limitation accounts of speech perception, is that informational masking increases perceptual and cognitive demands, thereby reducing the resources available for higher-level linguistic processing. Under this account, listeners may be less able to integrate semantic constraints with syntactic information during thematic-role assignment, resulting in reduced effects of animacy under informational masking. Alternatively, informational masking may increase uncertainty regarding the incoming speech signal, leading listeners to rely more heavily on semantic constraints to support sentence recognition. Under this cue-reliance account, listeners would be expected to place greater weight on animacy-based expectations when acoustic information becomes less reliable, resulting in enhanced animacy effects under informational masking. The present study was designed to test these competing hypotheses and to examine how informational masking influences the use of semantic constraints during recognition of syntactically complex speech.

To evaluate how informational masking influences the use of semantic constraints during recognition of syntactically complex speech, the present study examined both speech intelligibility and task-evoked pupil responses. Speech intelligibility provides a direct measure of recognition outcomes and has been widely used to examine the effects of background noise on speech perception (Bronkhorst, 2000; Mattys et al., 2012). Importantly, successful sentence repetition requires listeners not only to identify individual words but also to maintain linguistic information in working memory, assign thematic roles, and integrate information across the sentence before producing a response (Just and Carpenter, 1992; Gibson, 1998; Wingfield and Tun, 2007). Consequently, recognition accuracy reflects the combined outcome of perceptual, linguistic, and cognitive processes involved in sentence recognition. However, behavioral performance alone does not reveal the amount of processing effort required to achieve successful recognition, as listeners may reach similar levels of performance through different processing strategies or levels of cognitive resource allocation.

Task-evoked pupillometry provides a complementary measure of processing effort during speech perception. Previous research has demonstrated that pupil dilation is sensitive to perceptual challenge, linguistic complexity, and cognitive resource allocation during speech processing (Pichora-Fuller et al., 2016; Winn et al., 2018; Zekveld et al., 2018). In particular, pupillometry can reveal processing costs associated with successful speech recognition that are not evident from behavioral performance alone. Importantly, the pupil response unfolds continuously over time, providing an opportunity to examine the temporal dynamics of speech processing in addition to overall listening effort. Rather than reflecting a discrete processing stage, changes in pupil diameter are thought to index the continuous allocation of cognitive resources as listeners perceive, interpret, and integrate linguistic information throughout sentence processing (Zekveld et al., 2010; Wierda et al., 2012; Kuchinsky et al., 2016). Recent methodological advances in time-resolved analyses have made it possible to identify when linguistic information influences online speech processing, providing mechanistic insights beyond conventional summary measures such as peak or mean pupil dilation (van Rij et al., 2019; Johns et al., 2024).

Accordingly, examining intelligibility and the temporal dynamics of pupil responses together provides complementary information about both the outcome of speech recognition and the cognitive processes supporting successful recognition. Object-relative sentences provide a particularly informative context for examining the use of semantic constraints because their noncanonical syntactic structure places greater demands on thematic-role assignment and makes interpretation more sensitive to animacy information. For this reason, the present pupillometry analyses focused on object-relative sentences to test how informational masking influences the use of animacy-based semantic constraints under conditions of relatively high syntactic processing demand. GAMMs were used to characterize both the magnitude and temporal course of these semantic effects across masker conditions. Under a resource-limitation account, stronger informational masking should reduce listeners’ use of animacy-based semantic constraints, resulting in smaller animacy effects on sentence-recognition accuracy and less differentiation between animate and inanimate conditions in the pupil response. In contrast, under a cue-reliance account, stronger informational masking should increase reliance on animacy-based constraints, resulting in larger animacy effects on recognition accuracy and greater differentiation in the magnitude and/or temporal trajectory of pupil responses between animate and inanimate conditions.

## Methods

### Participants

Thirty-seven younger adults (mean age = 21.43 years, range = 18–32 years) with age-typical hearing participated in the study. Participants were assigned to the original- and reversed-speech masker groups in an alternating manner throughout data collection, resulting in 18 participants in the original-speech condition and 19 in the reversed-speech condition. Seventy-three percent of participants identified as female, 24% as male, and 3% as nonbinary. Seventy percent identified as White, 19% as Black/African American, 3% as Asian, and 8% as more than one race. All participants were native speakers of American English and had hearing thresholds within clinically normal limits (≤20 dB HL at 0.5, 1, 2, and 4 kHz), as assessed using conventional pure-tone air-conduction audiometry in each ear. Participants reported no history of neurological disorders, language disorders, or uncorrected visual impairments. Written informed consent was obtained prior to participation, and all procedures were approved by the Institutional Review Board at Temple University.

### Materials and stimuli

The sentence materials were adapted from DeDe (2015), which examined the influence of animacy on recognition of syntactically complex spoken sentences. The stimulus set consisted of subject-relative and object-relative sentences that varied in the animacy configuration of the critical noun phrases. These manipulations were designed to influence thematic-role assignment during sentence processing by altering the extent to which semantic expectations aligned with the syntactic structure of the sentence.

Example stimuli are shown below:

- OR-animate:
  *The director that the movie pleased received a prize at the film festival*.
- OR-inanimate:
  *The movie that the director watched received a prize at the film festival*.
- SR-animate:
  *The director that watched the movie received a prize at the film festival*.
- SR-inanimate:
  *The movie that pleased the director received a prize at the film festival*.

These sentence types were selected because successful recognition requires listeners to integrate syntactic and semantic information to assign thematic roles during online processing. Previous research has shown that animacy information influences processing of these sentence structures by providing semantic constraints that guide interpretation of “who did what to whom” during sentence recognition (Traxler et al., 2005). Critically, this work has shown that the processing demands associated with object relative sentences are mitigated when the sentential subject is inanimate rather than animate.

The experiment included 100 experimental sentences derived from 25 sentence sets, each recorded in four conditions crossing syntactic structure (subject-relative, object-relative) and semantic constraint condition (animate, inanimate critical noun phrase). To reduce predictability of sentence structure, the experimental items were combined with filler sentences representing multiple syntactic constructions, including parenthetical constructions, Garden Path sentences, and unrelated sentence types. Experimental and filler items were distributed across four stimulus lists containing 100 sentences each. Sentence sets and syntactic conditions were counterbalanced across lists, and presentation order was pseudorandomized such that no more than two consecutive trials originated from the same stimulus set.

All sentences were recorded by a female speaker of Standard American English in a sound-attenuated booth. Sentence durations were controlled across conditions, and individual recordings were segmented and edited to create separate audio files. Stimuli were filtered from 80 to 10,000 Hz and normalized using root-mean-square amplitude prior to masker mixing.

To manipulate informational masking, sentences were presented in either a two-talker speech masker or a time-reversed version of the same masker previously developed by Shen and Souza (2018). Masker type was manipulated between participants, with participants alternately assigned to the original and reversed speech masker conditions. A fixed target-to-masker ratio (TMR) of −5 dB was used in both masker conditions to maintain equivalent energetic masking while varying the availability of linguistic information in the masker. Stimuli were presented at 65 dB SPL and amplified individually using the National Acoustics Laboratories-Revised (NAL-R) linear prescription (Byrne et al., 2001) based on participants’ audiometric thresholds averaged across ears. This amplification procedure was implemented as part of a larger study that included both younger and older adults, although only younger-adult data are reported here.

### Procedure

Participants completed one stimulus list during each experimental visit. Visits were scheduled at least 1 week apart, and order of list presentation was counterbalanced across participants. The task was to repeat each sentence aloud immediately after presentation. Each session began with four practice trials (without feedback) to familiarize participants with the procedure.

Testing was conducted in a dimly illuminated double-walled sound booth. Auditory stimuli were delivered binaurally through Sennheiser HD-25 headphones. Pupil diameter was recorded using an EyeLink 1000 Plus eye tracker (SR Research, Ottawa, Canada) operating in remote mode with head stabilization and a sampling rate of 1,000 Hz. Pupil size was recorded from the left eye for 36 participants. For one participant, the right eye was recorded because a strong prescription in the left eye prevented reliable calibration; this exception was unrelated to experimental condition. Participants were seated approximately 58 cm from an LCD monitor. The display background was maintained at a constant gray level to minimize luminance-related fluctuations in pupil diameter, with luminance measured at 37 lux at the participant’s eye position.

The experiment was implemented in MATLAB R2018b (MathWorks, Natick, MA) using custom scripts and the EyeLink Toolbox (Cornelissen et al., 2002). Each trial began with presentation of a red fixation cross at the center of the screen. Following a 2,000-ms baseline period, the masker began, and the target sentence was presented 200 ms later. A 2,000-ms silent retention interval followed sentence offset to capture delayed pupil responses associated with sentence processing. At the end of this interval, the fixation cross changed from red to green, cueing participants to begin repeating the sentence aloud. After the participant completed the repetition, the experimenter initiated the next phase of the trial. A central gray box remained on the screen for 6,000 ms to allow pupil diameter to return toward baseline before the next trial. The trial-level procedure, including the timing of fixation, auditory presentation, retention interval, and response prompt, is illustrated in Figure 1. Participants were instructed to blink and rest their eyes during this interval. Pupil diameter was recorded continuously throughout testing, and all visual and auditory events were synchronized with time-stamped markers embedded in the data file.

**Figure 1:**
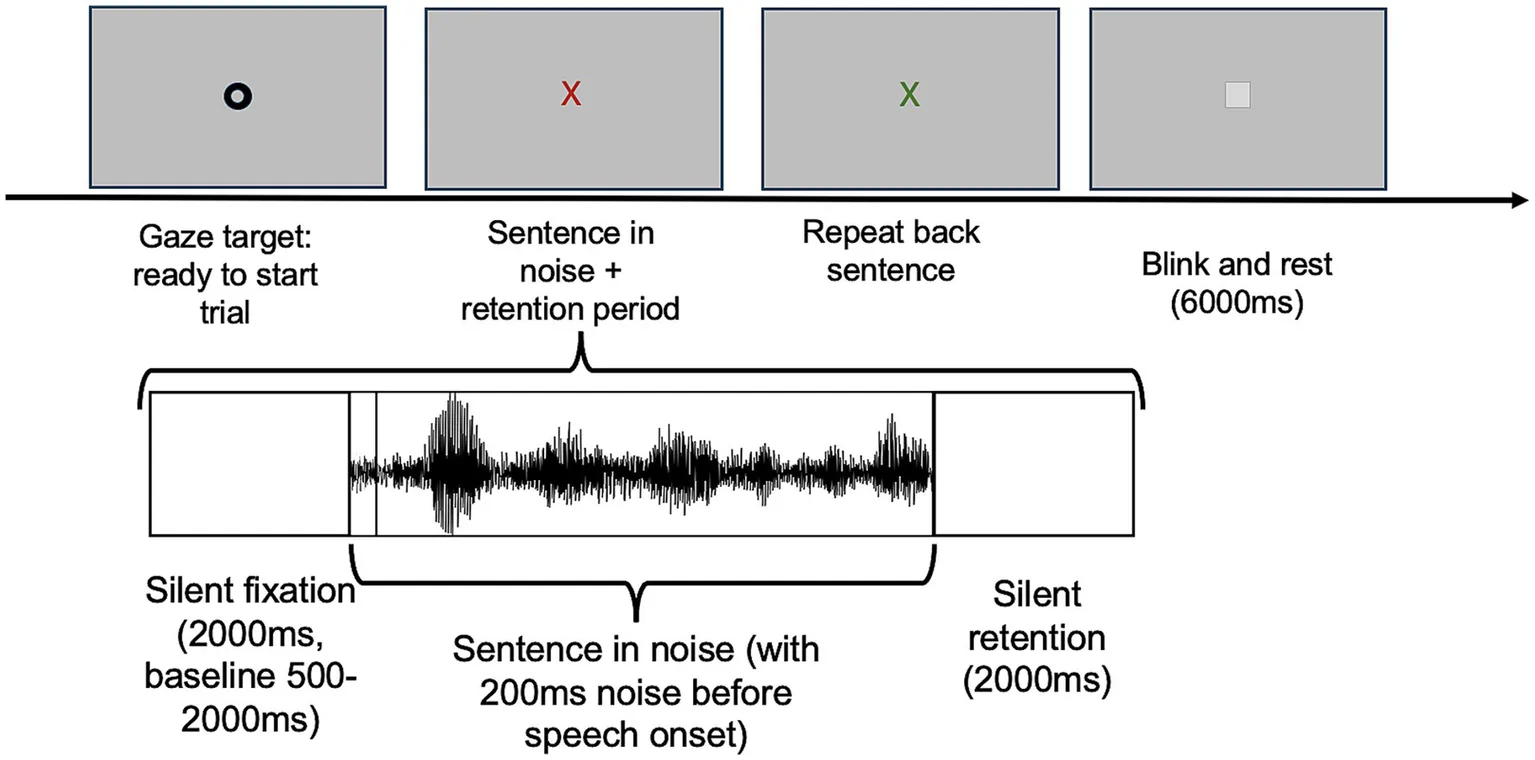
Schematic illustration of the sequence and timing of events within each trial of the pupillometry protocol.

Participant responses were audio-recorded and scored offline using a keyword-based scoring procedure. Keywords included the content words that conveyed the critical semantic information required for sentence interpretation, such as nouns and verbs, whereas function words (e.g., articles, prepositions, auxiliary verbs, and conjunctions) were excluded from scoring. Intelligibility was calculated as the percentage of correctly repeated keywords per sentence.

### Intelligibility data statistical analysis

Intelligibility was analyzed using generalized linear mixed-effects models (GLMMs) implemented in the lme4 package (Bates et al., 2014) in R version 4.4.1 (R Core Team, 2024). For each trial, the numbers of correctly and incorrectly repeated keywords were modeled using a binomial distribution with a logit link function. Fixed effects included syntactic structure (subject-relative, object-relative), semantic condition (animate, inanimate head noun), masker type (original, reversed), and experimental visit (1–4). All two-way and three-way interactions among syntactic structure, semantic condition, and masker type were included in the model. Categorical predictors were entered as treatment-coded factors, with object-relative sentences, inanimate head nouns, and the original speech masker serving as the reference levels. Visit number was entered as a continuous covariate to account for systematic changes in recognition performance across the four experimental visits.

Following current recommendations for mixed-effects modeling (Barr et al., 2013; Matuschek et al., 2017), we initially evaluated richer random-effects structures that included participant-specific random slopes for the within-subject predictors. These models produced singular fits, indicating that the data did not support estimation of the additional variance components. The final model therefore included random intercepts for participants and sentence items. Model estimation was performed using the bobyqa optimizer. Statistical significance of fixed effects was evaluated using Wald *z* tests. Significant interactions were followed by planned pairwise comparisons using estimated marginal means implemented in the emmeans package (Lenth, 2023). Statistical significance was evaluated using an alpha level of 0.05.

### Pupillometry processing and analysis

Raw EyeLink samples were reconstructed on the original 1,000-Hz timestamp timeline so that unavailable or omitted samples were represented explicitly as missing data. Samples were classified as invalid when horizontal gaze position, vertical gaze position, or pupil size was unavailable. Internal missing-data segments of ≤200 ms were linearly interpolated, consistent with established pupillometry preprocessing practices for brief periods of signal loss (Kret and Sjak-Shie, 2019), whereas longer gaps were retained as missing. Quality-control analyses indicated low levels of missing data. Across object-relative trials, mean raw missingness ranged from 1.58% to 2.40% across the four Semantic × Masker conditions. Following interpolation of gaps ≤200 ms, mean unresolved missingness ranged from 0.36 to 1.01%, with median unresolved missingness of 0% in all four conditions. Seven of 1,702 trials (0.41%) exceeded the 20% exclusion criterion and were removed. Exclusions were distributed across conditions as follows: Original/Inanimate, 1/414; Original/Animate, 1/414; Reversed/Inanimate, 2/437; and Reversed/Animate, 3/437. No participant was excluded because of missing pupil data.

The resulting pupil signal was smoothed using a 50-ms centered moving average (Mathôt et al., 2018; Winn et al., 2018), with unresolved gaps retained rather than bridged by smoothing. Baseline pupil size was calculated from 500 to 2,000 ms following the recorded baseline-onset marker. Trials with more than 20% unresolved missing samples within the 0–6,000-ms post-speech-onset analysis interval were excluded from the pupil analysis.

Pupil responses were expressed as percentage change relative to baseline to reduce between-participant variability in absolute pupil size and facilitate comparison of task-evoked pupil responses across listeners. The baseline-normalized data were subsequently averaged into nonoverlapping 100-ms time bins for statistical analysis. To provide a common reference at sentence onset while reducing sensitivity to an isolated missing sample, each trial was additionally centered by subtracting the mean normalized pupil response during the first 400 ms following sentence onset from all time bins within that trial.

Because the primary objective was to characterize the influence of semantic constraints under conditions of high syntactic processing demand, pupillometry analyses were restricted to object-relative sentences, which have consistently been shown to exhibit robust animacy effects in previous psycholinguistic research (Traxler et al., 2002; DeDe, 2015). Analyses were restricted to the interval from sentence onset to 6,000-ms after sentence onset. Pupil responses were analyzed using generalized additive mixed models (GAMMs) implemented in the mgcv package (Wood, 2017). GAMMs were selected because they flexibly model nonlinear changes in pupil responses over time without requiring a predefined response shape. Fixed effects included semantic condition, masker type, and their interaction. A smooth term modeled the overall time course of the pupil response, while difference smooths estimated time-varying deviations associated with semantic condition, masker type, and their interaction. Factor-smooth interactions were included for participant and sentence item to account for participant- and item-specific temporal variability. Models additionally incorporated an AR(1) error structure to account for residual temporal autocorrelation with ρ = 0.869, estimated from lag-1 residual autocorrelation in a preliminary model without AR correction. Model comparisons were performed sequentially to evaluate the contributions of semantic condition, masker type, and their interaction to pupil responses. The full GAMM was specified as below. Here, SemanticAnim, NoiseOrig, and AnimOrig were ordered-factor indicators used to estimate the semantic-condition, masker-condition, and Semantic × Masker difference smooths, respectively.

pupil_final ~ Semantic * Noise + s(timeos, k = 10) + s(timeos, by = SemanticAnim, k = 10) + s(timeos, by = NoiseOrig, k = 10) + s(timeos, by = AnimOrig, k = 10) + s(timeos, subject, bs = “fs,” k = 5) + s(timeos, Item, bs = “fs,” k = 5).

## Results

### Intelligibility results

The generalized linear mixed-effects model revealed a significant main effect of semantic condition (*β* = −0.65, SE = 0.08, *z* = −8.59, *p* < 0.001), a significant main effect of syntactic structure (β = −0.26, SE = 0.08, *z* = −3.38, *p* < 0.001), and a non-significant main effect of masker type (β = 0.27, SE = 0.31, *z* = 0.86, *p* = 0.390). A significant Syntactic Structure × Semantic Condition interaction was also observed (β = 0.72, SE = 0.11, *z* = 6.70, *p* < 0.001), as was a significant Syntactic Structure × Masker Type interaction (β = 0.27, SE = 0.11, *z* = 2.37, *p* = 0.018). Neither the Semantic Condition × Masker Type interaction (*p* = 0.369) nor the three-way interaction among syntactic structure, semantic condition, and masker type (*p* = 0.923) reached significance. Recognition accuracy improved across visits (β = 0.25, SE = 0.02, *z* = 13.98, *p* < 0.001), indicating a positive linear association between visit number and performance. The final model coefficients are presented in Table 1, and observed accuracy for each experimental condition is shown in Figure 2.

**Table 1:** Fixed effects from the generalized linear mixed-effects model predicting keyword recognition accuracy.

Fixed effect	*β*	SE	z	*p*
Intercept	1.204 (0.694–1.713)	0.260	4.63	<0.001
Syntactic structure	−0.263 (−0.414 to −0.110)	0.078	−3.38	<0.001
Semantic condition	−0.647 (−0.794 to −0.499)	0.075	−8.59	<0.001
Masker	0.272 (−0.347–0.891)	0.316	0.86	0.390
Visit	0.247 (0.212–0.282)	0.018	13.98	<0.001
Syntactic × Semantic	0.717 (0.506–0.926)	0.107	6.70	<0.001
Syntactic × Masker	0.270 (0.046–0.493)	0.114	2.37	0.018
Semantic × Masker	0.098 (−0.115 to 0.311)	0.109	0.90	0.369
Syntactic × Semantic × Masker	0.015 (−0.294 to 0.325)	0.158	0.10	0.923

Treatment coding was used with Object-relative, Inanimate, and Original masker as the reference levels. Random intercepts were included for participants and sentence items. β coefficients and 95% confidence intervals are reported on the log-odds scale.

**Figure 2:**
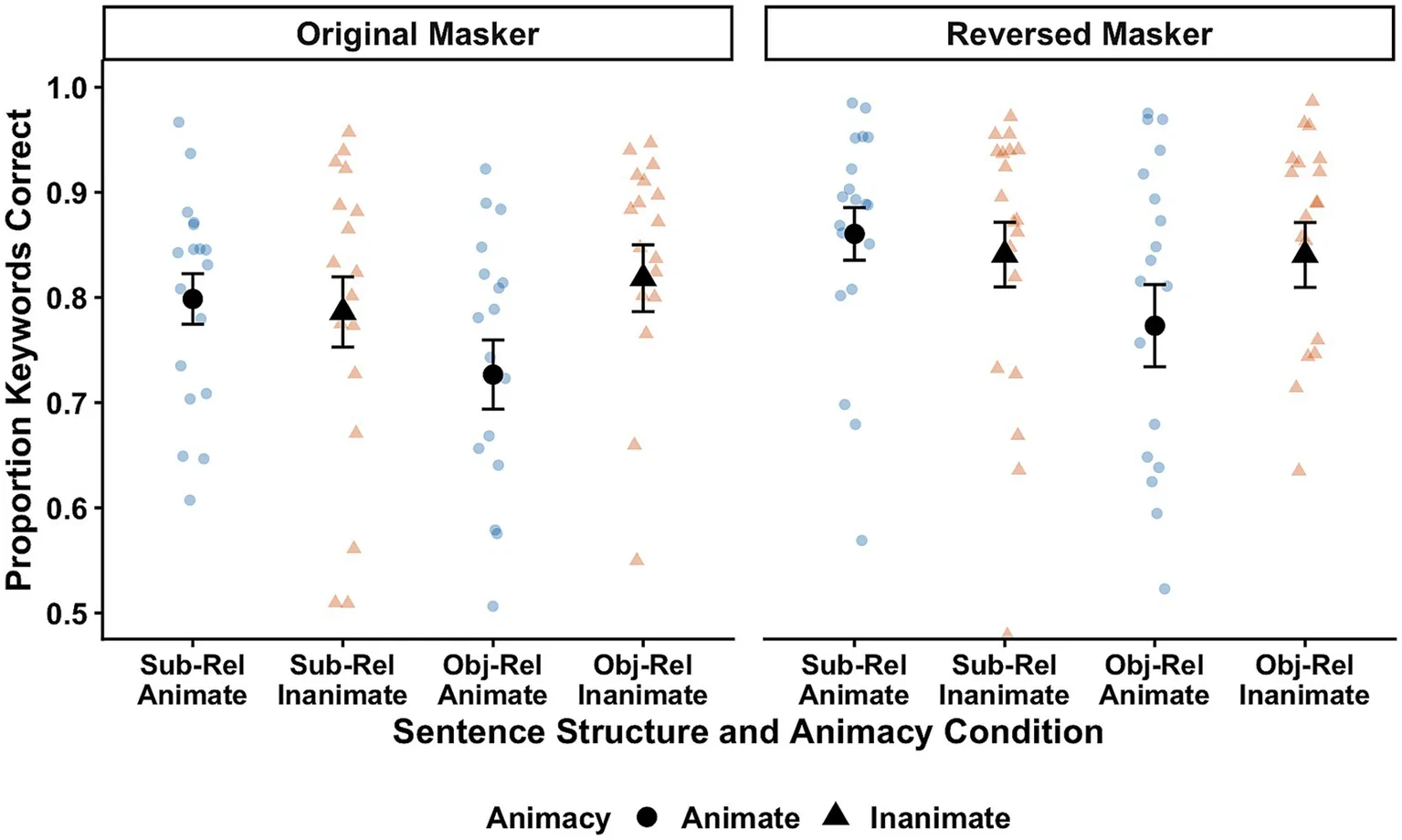
Mean keyword recognition accuracy for subject-relative (Sub-Rel) and object-relative (Obj-Rel) sentences as a function of semantic condition (animate, inanimate) under the original speech masker and reversed speech masker conditions. Points represent individual participants, black symbols indicate condition means, and error bars denote ±1 standard error of the mean.

To facilitate interpretation of the significant Syntactic Structure × Semantic Condition interaction, estimated marginal means are presented in Table 2. For object-relative sentences, predicted recognition accuracy was consistently higher for inanimate than animate sentences under both masker conditions. In contrast, only small differences between semantic conditions were observed for subject-relative sentences. These estimated marginal means illustrate that semantic constraints had a substantially larger influence on recognition of object-relative than subject-relative sentences.

**Table 2:** Estimated marginal means (predicted recognition accuracy) from the generalized linear mixed-effects model.

Sentence structure	Semantic condition	Original masker	Reversed masker
Object-relative	Inanimate	86.1% (78.9–91.1)	89.0% (83.2–93.0)
Object-relative	Animate	76.4% (66.3–84.2)	82.4% (74.1–88.5)
Subject-relative	Inanimate	82.6% (74.2–88.7)	89.1% (83.3–93.1)
Subject-relative	Animate	83.6% (75.5–89.4)	90.8% (85.7–94.2)

Values are estimated marginal means (predicted probabilities) with 95% confidence intervals.

### Pupillometry results

Pupillometry analyses were restricted to object-relative sentences because the primary objective was to characterize the influence of animacy-based semantic constraints under conditions of high syntactic processing demand. Table 3 reports GAMM model comparison results. Because these were prespecified sequential model comparisons rather than *post hoc* pairwise tests, no multiple-comparison correction was applied. Sequential model comparisons indicated that adding semantic condition significantly improved model fit relative to the baseline model (ΔAIC = −50.3, ΔDeviance = 53.43, *p* < 0.001). Adding masker type did not yield a significant improvement in the sequential deviance comparison (*p* = 0.535), although AIC decreased by 18.4 points. In contrast, adding the Semantic × Masker interaction substantially improved model fit (ΔAIC = −231.8, ΔDeviance = 236.53, *p* < 0.001), indicating that the influence of semantic condition on pupil responses depended on masker type. Figure 3 shows population-level GAMM-predicted pupil trajectories for animate and inanimate sentences under the two masker conditions.

**Table 3:** Sequential comparisons of generalized additive mixed models of normalized pupil responses.

Comparison	Added predictor(s)	ΔAIC	ΔDeviance	Δdf	*p*
M1 vs. M0	Semantic condition	−50.3	53.43	1.82	<0.001
M2 vs. M1	Masker type	−18.4	−5.02	−5.94	0.535
M3 vs. M2	Semantic × Masker	−231.8	236.53	2.31	<0.001

ΔAIC is calculated relative to the immediately preceding model; negative values indicate lower AIC. Degrees of freedom are effective degrees of freedom from penalized GAMMs and therefore need not increase monotonically across models.

**Figure 3:**
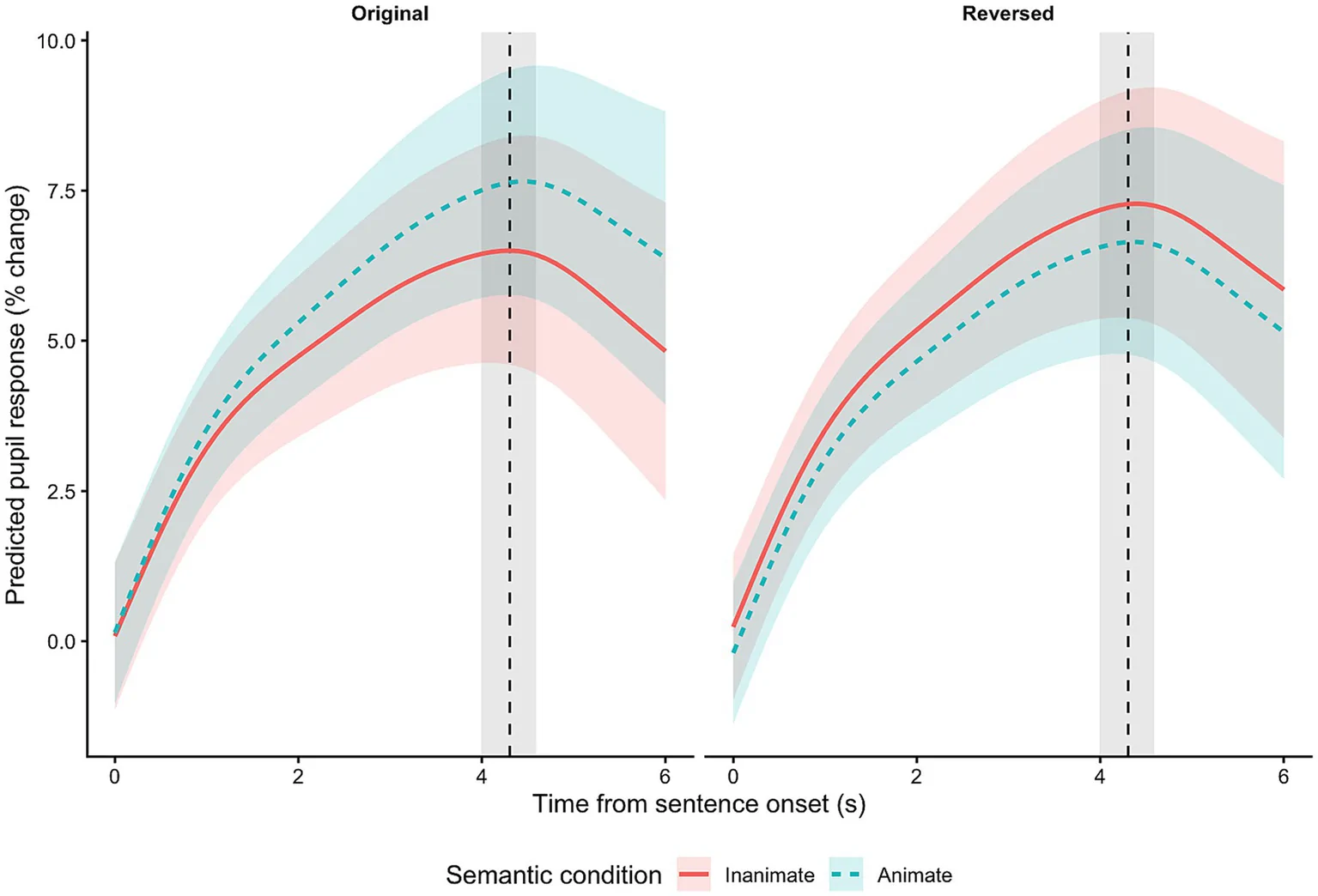
GAMM-predicted pupil-response trajectories for animate and inanimate object-relative sentences under the original-speech masker (left) and reversed-speech masker (right) conditions. Shaded ribbons represent 95% confidence intervals. The vertical dashed line denotes the mean sentence offset across trials, and the gray shaded region indicates the interquartile range (IQR) of sentence offsets. Population-level predictions are shown over the full 0–6000 ms analysis window, with participant- and item-specific smooths excluded from the displayed estimates.

In GAMM models, parametric effects capture overall differences in pupil-response magnitude across conditions, whereas smooth terms capture differences in how pupil responses change over time. The final GAMM revealed a significant parametric effect of semantic condition (*β* = −0.57, SE = 0.23, *t* = −2.51, *p* = 0.012) and a significant Semantic × Masker interaction (*β* = 1.35, SE = 0.32, *t* = 4.19, *p* < 0.001). The parametric effect of masker type was not significant (*β* = −0.57, SE = 0.46, *t* = −1.24, *p* = 0.216). With Inanimate sentences and the Reversed masker serving as reference levels, the negative semantic coefficient indicated lower pupil responses for Animate than Inanimate sentences in the Reversed condition, whereas the positive interaction coefficient indicated that this semantic effect differed substantially in the Original masker.

Examination of the smooth terms showed a significant nonlinear change in pupil response over time (*edf* = 6.68, *F* = 13.99, *p* < 0.001). However, the time-varying effect of semantic condition was not significant (*edf* = 1.01, *F* = 0.19, *p* = 0.675), nor was the time-varying effect of masker type (*edf* = 1.01, *F* = 0.70, *p* = 0.399). The Semantic × Masker difference smooth also did not reach significance (*edf* = 1.03, *F* = 3.68, *p* = 0.056). Thus, the results provide evidence that masker type modulated the magnitude and direction of the semantic effect on pupil responses, but not clear evidence that it altered the temporal shape of that effect.

Figure 4 displays the model-estimated semantic effect (Animate − Inanimate) separately for the Original and Reversed masker conditions. Plotting the two difference curves on the same axes provides a direct visualization of the Semantic × Masker interaction, with the separation between the curves reflecting the masker-dependent difference in the magnitude and direction of the semantic effect. In the Original masker, the semantic effect was positive, indicating relatively larger pupil responses for Animate than Inanimate sentences, whereas the effect was negative in the Reversed masker. Although the two curves differed in overall magnitude and direction, the corresponding time-varying Semantic × Masker smooth did not reach significance. Thus, Figure 4 illustrates the masker-dependent semantic effect but should not be interpreted as identifying specific time periods in which the interaction was statistically significant. The present sentence-onset-aligned GAMM therefore supports a difference in the overall semantic modulation of pupil responses across masker conditions, while providing limited evidence that the temporal trajectory of that modulation differed systematically over time. Future event-aligned analyses may be better suited to determining whether specific linguistic events contribute to these masker-dependent effects.

**Figure 4:**
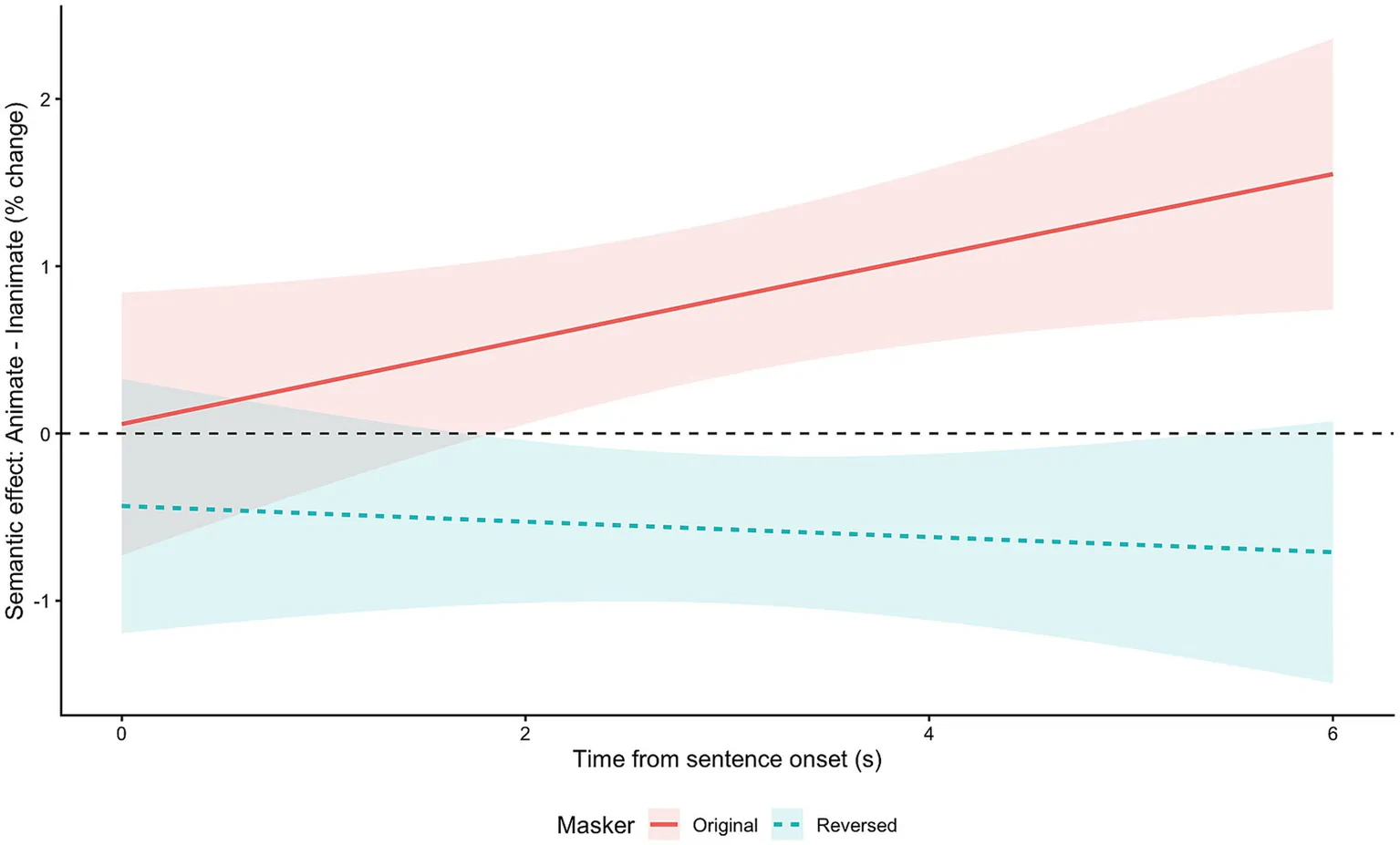
Model-estimated semantic effects on normalized pupil responses under the original- and reversed-speech masker conditions. Difference curves represent the estimated animate − inanimate contrast over time for each masker condition, with shaded regions indicating 95% confidence intervals. The separation between the two curves illustrates the masker-dependent semantic effect. Estimates are shown over the full 0–6000 ms analysis window.

## Discussion

### Semantic effects during syntactically complex speech perception

The behavioral findings first replicated previous studies in showing that semantic animacy constraints influence the comprehension of object-relative sentences (Traxler et al., 2002; DeDe, 2015). Consistent with previous studies, the effect of semantic condition was substantially greater for syntactically complex object-relative sentences than for subject-relative sentences. Although only small differences between semantic conditions were observed for subject-relative sentences, the effect was considerably larger for object-relative sentences, indicating that semantic constraints become increasingly important as syntactic processing demands increase. Because the present task required listeners to recognize and immediately repeat entire sentences, these findings further suggest that semantic information supports not only online sentence interpretation but also the successful encoding and maintenance of syntactically complex speech for subsequent recall.

The present findings extend previous work in several important ways. Previous studies demonstrating animacy effects in object-relative sentences have primarily employed reading paradigms or spoken language tasks under relatively favorable listening conditions. The present behavioral results show that semantic facilitation persists during spoken sentence recognition and immediate repetition in the presence of competing speech, indicating that listeners continue to benefit from semantic constraints under adverse listening conditions. Importantly, the accompanying pupillometry findings further suggest that comparable levels of behavioral performance may be achieved through different patterns of cognitive resource allocation, highlighting the value of combining behavioral and physiological measures to characterize speech processing under challenging listening conditions.

Constraint-based accounts of sentence processing propose that listeners continuously integrate syntactic and semantic information during comprehension, allowing semantic plausibility to constrain thematic-role assignment and reduce processing uncertainty (MacDonald et al., 1994; Trueswell et al., 1994; Traxler et al., 2002). Under adverse listening conditions, such semantic constraints may become particularly important as listeners compensate for degraded acoustic input (Rönnberg et al., 2013; Pichora-Fuller et al., 2016). The present intelligibility results are consistent with this view, suggesting that semantic plausibility serves as an important source of information during the processing of syntactically challenging spoken sentences. The larger animacy effects observed for object-relative sentences suggest that semantic constraints become increasingly important as syntactic processing demands increase. Together with the pupillometry findings, these results support the broader hypothesis that listeners dynamically adjust their reliance on higher-level linguistic information as task demands increase.

### Animacy influences the magnitude of pupil responses across masker conditions

While the intelligibility results replicated previous findings (Traxler et al., 2002; DeDe, 2015), the pupillometry data provide new insight into how semantic information shapes cognitive processing during speech perception in background noise. Pupil dilation is commonly used as an indirect physiological index of cognitive resource allocation during listening, although it does not provide a direct measure of listening effort or identify the specific cognitive processes underlying changes in pupil dynamics. Accordingly, we interpret the present pupil effects as reflecting differences in processing demands within the framework established by previous pupillometry research. The GAMM analyses revealed a significant interaction between semantic condition and masker type in the magnitude of pupil responses. In the original-speech masker, animate object-relative sentences were associated with larger pupil responses than inanimate sentences, whereas the semantic effect was reversed under the reversed-speech masker, with animate sentences associated with smaller pupil responses than inanimate sentences. The corresponding time-varying interaction smooth did not reach significance, providing no clear evidence that masker type altered the temporal shape of the semantic effect. Thus, informational masking appeared to influence the magnitude and direction of semantic modulation rather than producing a robust difference in its temporal trajectory.

Previous pupillometry studies of speech perception have commonly interpreted larger task-evoked pupil responses as an index of increased listening effort or cognitive processing demands (Zekveld et al., 2010; Winn et al., 2015). Within this framework, the present findings suggest that semantic constraints are associated with dynamic changes in the allocation of cognitive resources during sentence processing, with the clearest effect reflected in how the magnitude of semantic modulation differed across masker conditions. Under the original-speech masker, animate object-relative sentences elicited larger pupil responses than inanimate sentences, whereas under the reversed-speech masker the semantic contrast shifted toward relatively larger responses for inanimate sentences. This pattern suggests that the processing consequences of animacy cues depend on the nature of competing speech rather than reflecting a fixed processing advantage or disadvantage associated with animacy itself. One possibility is that intelligible competing speech changes the extent to which listeners rely on semantic constraints during sentence interpretation, thereby altering the processing demands associated with animate and inanimate sentence structures. This interpretation is consistent with the cue-reliance account, in which greater informational interference may increase reliance on available semantic constraints. However, because pupil dilation does not identify the specific cognitive operation responsible for the effect, the present data cannot determine whether the interaction reflects differences in thematic-role assignment, semantic integration, conflict resolution, or other aspects of sentence processing.

This interpretation is consistent with contemporary views of sentence processing as a dynamic process in which semantic and syntactic information are continuously integrated over time (MacDonald et al., 1994; Traxler et al., 2002). At the same time, the present results provide limited evidence that the Semantic × Masker interaction itself changed systematically over the course of the sentence. The interaction was primarily expressed in the magnitude and direction of the semantic effect, whereas the time-varying interaction smooth did not reach significance. Thus, although pupil responses remain useful for characterizing how processing demands unfold during sentence recognition (Wierda et al., 2012; Johns et al., 2024), the present findings should not be interpreted as identifying discrete temporal stages at which informational masking alters semantic processing. Event-aligned analyses that relate pupil responses to specific linguistic landmarks may be better suited to determining when particular semantic and syntactic processes contribute to the observed masker-dependent effects.

### Informational masking modulates semantic cue use

Previous studies of informational masking have often reported larger pupil responses for competing speech maskers than for less informationally demanding maskers, suggesting increased cognitive demands during speech perception (Zekveld et al., 2010; Koelewijn et al., 2012). In the present study, however, masker type did not produce a significant overall effect on pupil responses. Instead, its influence depended on semantic condition, as indicated by the significant animacy-by-masker interaction. Specifically, the original speech masker produced a larger and directionally different animacy effect than the reversed speech masker, indicating that informational masking altered how semantic constraints contributed to processing demands rather than uniformly increasing pupil dilation across sentence types.

This pattern may reflect important differences in the linguistic demands of the present task. Many previous informational masking studies have employed isolated words, matrix sentences, or relatively simple sentence materials in which successful performance depends primarily on target-masker segregation and accurate speech identification. In contrast, listeners in the present study recognized and repeated syntactically complex object-relative sentences, requiring not only accurate perception of the acoustic signal but also online thematic-role assignment and semantic integration. Although the task involved sentence repetition rather than an explicit comprehension judgment, these higher-level linguistic processes are engaged as part of online spoken sentence processing (MacDonald et al., 1994; Friederici, 2002). Consequently, the greater linguistic demands of the present materials may have increased the opportunity for listeners to draw on semantic information, helping to explain why informational masking modulated the animacy effect rather than producing a uniform increase in pupil responses.

The pupillometry findings suggest that informational masking influences how semantic information contributes to processing demands during sentence recognition rather than simply increasing overall listening effort. The significant animacy-by-masker interaction indicates that the influence of semantic constraints differed between the original and reversed speech masker conditions, with a larger and directionally different animacy effect under the original speech masker. This pattern favors the cue-reliance account over the resource-limitation hypothesis proposed here. In Norman and Bobrow’s (1975) framework, the present listening conditions may still have fallen within a resource-limited range in which additional cognitive resources could be recruited to support performance, rather than a data-limited range in which further allocation would provide little benefit. For these younger adults, the −5 dB TMR condition may therefore have increased processing demands without exhausting available resources, allowing greater reliance on semantic constraints under informational masking. Aging and hearing loss may shift listeners toward more constrained processing conditions, however, by increasing perceptual demands and reducing the resources available for higher-level language processing (Ayasse and Wingfield, 2018).

More broadly, the present findings also highlight that behavioral performance and pupil responses provide related but non-equivalent information about processing. Although inanimate object-relative sentences were recognized more accurately than animate object-relative sentences, greater pupil dilation does not necessarily imply poorer performance, and the reversal of the pupil-based animacy effect across masker conditions further indicates that processing demand and recognition accuracy need not vary in parallel.

### Limitations and future directions

Several limitations should be considered when interpreting the present findings. First, the study was conducted exclusively in young adults with normal hearing. Because the use of semantic cues and the allocation of cognitive resources during listening may differ across the lifespan and in individuals with hearing loss, it remains unclear whether the observed effects generalize to older listeners or clinical populations. Given that older adults often rely more heavily on contextual information during speech perception (DeDe, 2015), the influence of animacy on processing demands may be even more pronounced in these populations.

Second, the masker manipulation was implemented as a between-subjects factor, with each participant experiencing only one masker condition. This design minimized potential learning and adaptation effects associated with repeated exposure to the sentence materials but limited the ability to directly compare individual listeners across masker conditions. Given the relatively modest number of participants in each masker group, the study had limited sensitivity to smaller between-subjects effects. Although the primary analyses incorporated repeated observations across sentences and modeled participant- and item-level variability, between-participant variability in physiological responses or listening strategies may nevertheless have contributed to the observed masker-dependent differences. Future studies with larger samples or within-subject manipulations of masker type would provide a more sensitive test of how informational masking influences pupil responses and the deployment of semantic cues during sentence comprehension.

Third, the present study focused on phasic pupil responses elicited by the unfolding sentence. An emerging direction in pupillometry research is to examine baseline pupil diameter as an index of listeners’ preparatory attentional state, arousal, and mobilization of cognitive resources before speech onset (Ayasse and Wingfield, 2020; Relaño-Iborra et al., 2022; Johns et al., 2024). Future work should determine whether baseline pupil measures predict subsequent reliance on semantic constraints under informational masking, thereby linking tonic and phasic components of the pupillary response during spoken language processing.

Finally, the present analyses demonstrate that the magnitude and direction of semantic modulation of pupil responses depend on masker condition, but provide limited evidence that these effects differ systematically in their temporal trajectory. Future work using more fine-grained time-resolved approaches, such as temporal response functions or event-based analyses aligned to critical linguistic regions, may help determine when semantic and syntactic information influence processing during the unfolding sentence. Such approaches may further clarify the mechanisms underlying the masker-dependent use of semantic constraints during spoken language recognition.

## Conclusion

Despite these limitations, the present findings provide new insight into how semantic information supports spoken sentence recognition under adverse listening conditions. Consistent with previous research, semantic effects were greatest for syntactically complex object-relative sentences, demonstrating that listeners continue to draw on animacy-based semantic constraints in the presence of competing speech. More importantly, the pupillometry findings showed that semantic condition interacted with masker type, indicating that informational masking altered the magnitude and direction of semantic modulation of pupil responses rather than uniformly increasing processing demands. Together, these findings suggest that competing linguistic information changes how semantic constraints contribute to cognitive resource allocation during sentence recognition. More broadly, they highlight the value of combining behavioral and pupillometric measures to characterize how linguistic and perceptual demands jointly shape spoken language processing and provide a foundation for future event-aligned studies of the mechanisms underlying these effects.

## Data Availability

The raw data supporting the conclusions of this article will be made available by the authors, without undue reservation.
